# Nonsecretory multiple myeloma

**DOI:** 10.4103/0019-5413.55979

**Published:** 2009

**Authors:** Seshikanth Middela, Prakash Kanse

**Affiliations:** Department of Orthopedics, Withybush General Hospital, UK

**Keywords:** Multiple myeloma, non secretory, bone tumor

## Abstract

Multiple myeloma is characterized by clonal proliferation of plasma cells usually of the B cell type. The skeletal manifestations are usually osteolytic lesions whose differential diagnosis includes primary and secondary bone tumor. This tumor is characterized by the presence of abnormal paraprotein 8 in blood and urine. However, one to five per cent of the cases do not have any protein. Hence they are termed nonsecretory. It often poses a diagnostic dilemma when it is presented to orthopedic surgeons with no clear features of the disease. Our case report exemplifies such a diagnostic dilemma. A high index of suspicion must be borne in mind when excluding multiple myeloma as a cause of pain, pathological fracture or lytic lesion.

## INTRODUCTION

Multiple myeloma is a malignant proliferation of plasma cells within the bone marrow. It is the most common primary malignant tumor of the bone, about 27% of the biopsied bone tumors.^1^ Measurement of circulating monoclonal immunoglobulin has been the standard for diagnosis, prognosis and management. However, in about one to five per cent of multiple myeloma cases no protein can be detected^2^ and these patients are known to have a nonsecretory type of myeloma. Diagnosis in these cases then depends upon bone marrow biopsy and subsequent demonstration of plasmocytes. A search for “non-secretory multiple myeloma” and “nonsecretory multiple myeloma,” in the PubMed, yielded less than 80 case reports since 1972. These patients are often a diagnostic dilemma and a high index of suspicion should be borne in mind, especially in a patient with osteolytic lesions. This uncommon presentation may lead to delay in diagnosis if presented to orthopedic surgeons. We present a case report of a patient who presented to District General Hospital with back pain and was subsequently diagnosed with nonsecretory multiple myeloma.

## CASE REPORT

A 60-year-old male was referred to our clinic with complaints of low back ache of one year duration. The pain was radiating down the right lower limb as far as the knee joint. There was no history of trauma or history suggestive of any significant medical co-morbid conditions.

Examination revealed tenderness on the right side of the lumbosacral spine and right sacroiliac joint. There was para vertebral muscle spasm with loss of lumbar lordosis. Hip movements were normal with no major disability. There were no root stretch signs and no neurological deficit was noted.

**Figure 1:**
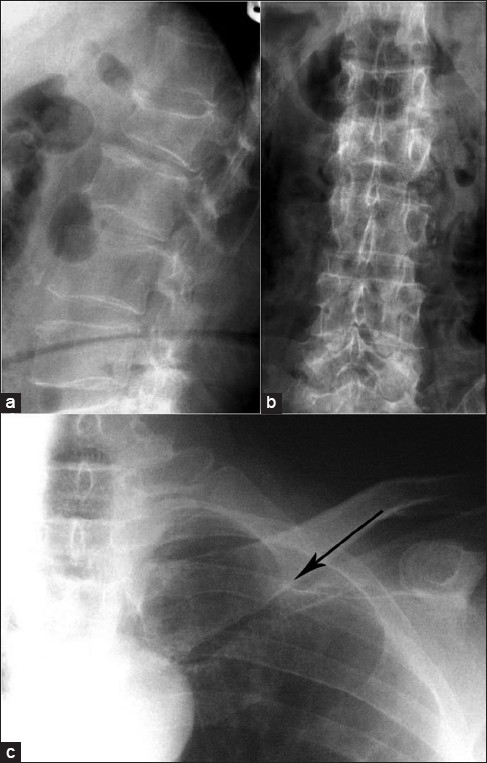
X-ray lumbosacral spine lateral view (a) and anteroposterior view (b) showing compression fracture of L2 vertebrae. X-ray of (L) shoulder with clavicle shows a thin hair line fracture at the medial end of left clavicle (solid arrow)

**Figure 2:**
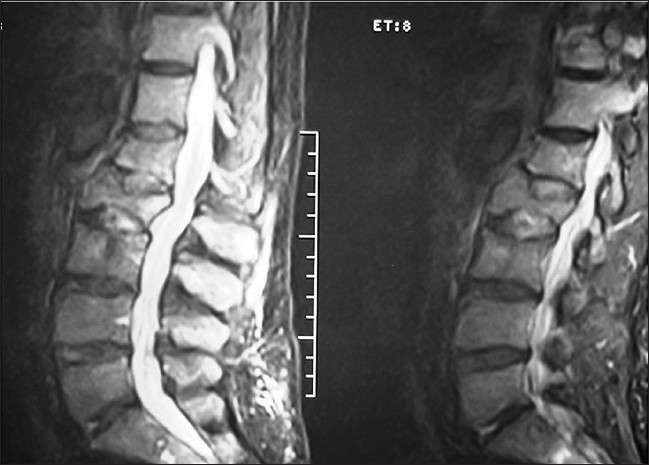
T2W1 of MRI of Lumbasacral spine shows compression fracture

**Figure 3:**
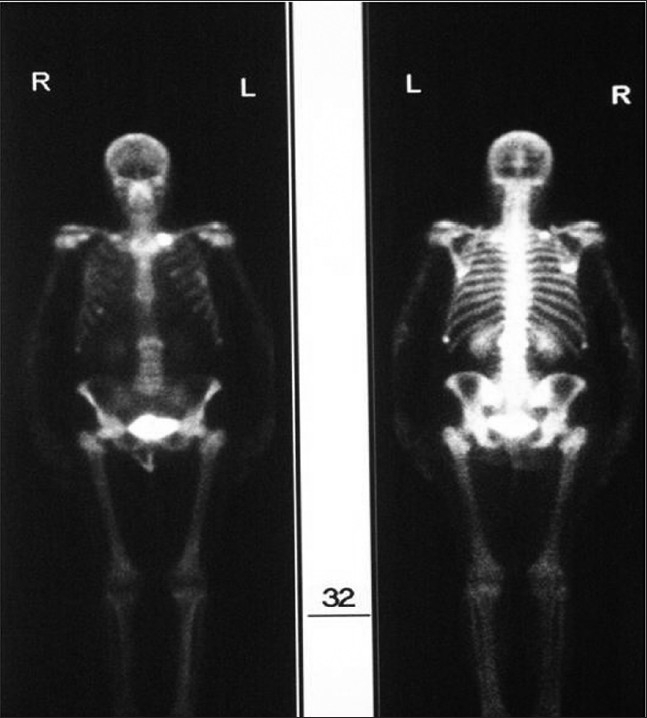
Bone scan showing increased uptake at the medial end of the left clavicle

An x-ray of the lumbosacral spine showed evidence of old fracture of L1 vertebra [Figure 1]. The Magnetic Resonance Imaging (MRI) scan showed collapse of the L2 vertebra with extrusion of the bone posteriorly into the spinal canal [Figure 2]. There was soft tissue infiltration seen in the sacrum. While awaiting clinic review the patient presented to the casualty with fracture clavicle on the left side following a trivial fall. The fracture of the clavicle was at the medial end (unusual site). A subsequent bone scan showed increased uptake in the medial end of the left clavicle and L2 vertebra. There were areas of increased activity in the region of the ribs [Figure 3].

Lab investigations revealed hemoglobin of 12.8 g/dl, erythrocyte sedimentation rate (ESR) of 21mm/hr and hematocrit ratio of 0.357 (normal 0.370-0.470 ratio). Creatinine was 50 *μ*mol/l (normal 50-141*μ*mol/l) and urea of 5.9mmol/l (normal 2.5-7.5 mmol/l). The serum calcium 2.28 mmol/l (normal 2.1-2.6 mmol/l) and phosphate levels 1.13 mmol/l (normal 0.81-1.45 mmol/l) were normal. Lactic acid dehydrogenase was elevated, 1061 IU/l (normal 313-618 IU/l) and Alkaline phosphatase was normal, 125 IU/l (normal 25-150 IU/l).

Myeloma screen which did not reveal presence of any abnormal compact bands was obtained and there was no Bence-Jones proteinuria. The levels of the immunoglobulin were Ig G 6.6 g/l (normal 5.3-16.5 g/l), Ig A 0.21 g/l (normal 0.80-4.00 g/l) and Ig M was 0.36 g/l (normal 0.50-2.00 g/l), which were lower than normal.

**Figure 4:**
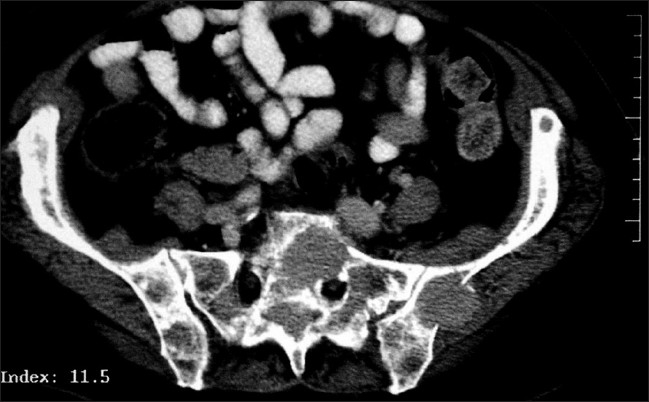
CT scan showing soft tissue infiltration into the left sacrum

In view of the bony pain, pathological fracture of the L2 vertebra, unusual fracture of the clavicle, normal myeloma screen, normal calcium levels and bone scan findings it was decided to organize a Computerized Tomography (CT) scan of the thorax and abdomen to look for the primary with secondaries as the first working diagnosis. The CT scan merely confirmed the findings of MRI scan and there was no evidence of any primary or other secondaries [Figure 4]. A bone biopsy was planned.

A percutaneous bone biopsy from the medial end of the clavicle was done because of easy accessibility which was negative for malignancy. A CT guided biopsy from the L2 vertebra was again negative for malignancy. A bone marrow aspirate and trephine biopsy were done from the iliac crest which confirmed the diagnosis of multiple myeloma. The biopsy showed a hyper cellular marrow with normal haemopoiesis replaced entirely with malignant plasma cells suggestive of myeloma. Aspirate showed plasma cells to be about 52% of the marrow with marked atypia. The patient was referred to further treatment in the form of chemotherapy and radiotherapy. The patient died one month after diagnosis while undergoing treatment. There was, however, a delay of two months between the first presentation and final diagnosis.

## DISCUSSION

Multiple myeloma is a disorder of the bone marrow which accounts for 10-15% of all blood cancers and one to two per cent of all malignancies.^3^,^4^ In the United Kingdom it accounts for 6.6 per 100,000.^5^ Among the varied presentation 10-40% is asymptomatic and 50-70% will have bony pain due to lytic lesions and pathological fractures.^3^ A high index of suspicion should be kept in mind to avoid diagnostic delay.

These tumors are characterized by proliferation of malignant plasma cells in the bone marrow and often associated with the production of monoclonal immunoglobulin (M-component) secreted in blood and urine. The protein is often detected by serum electrophoresis. In about one to five per cent of cases these may not be demonstrated and these cases are termed as nonsecretory multiple myeloma.^2^,^6^,^7^

Nonsecretory multiple myelomas were first described in 1958 by Serre.^8^ Since then numerous case reports have appeared describing variations in microscopic appearances^9^–^11^ of the tumor. It has been postulated that there may be reduced protein synthesis or increase in breakdown of abnormal immunoglobulin chains intracellular or extracellular. Immunoglobulin is synthesized but not secreted possibly due to reduced permeability or absence and alteration of intracellular transport of the light chains. It may mean that there may be intermittent excretion of immunoglobulin evading detection.^2^,^9^,^12^,^13^

Some researchers have further classified the nonsecretory myeloma based on the finding of intracytoplasmic immunoglobulin. They separated them into two types - nonproducer type (about 15%) where immunoglobulin was not found in plasma cells and in the remaining 85% called producer type the immunoglobulin is demonstrable in plasma cells but not in blood.^9^,^14^–^16^ Whether this is of any prognostic significance remains to be proved in the absence of large volume of cases.^9^

The above patient is a classic case of nonsecretory myeloma and meets the criteria laid down by the international myeloma working group.^17^ It was also a diagnostic dilemma as other disorders such as secondaries, osteoporosis and hyperparathyroidism can present with a similar picture.^16^ Our patient did not undergo serum immunoglobulin-free light chain assay (FLC). Patients with nonsecretory myeloma seem to have less incidence of renal insufficiency presumably because light chains are not being secreted in the urine.^2^ Once diagnosed, treatment remains the same as for multiple myeloma. The response to treatment and the prognosis remains the same.^6^,^9^,^17^ Arguments have been placed in earlier reports about patients with nonsecretory myeloma having better survival rates because of their early presentation and absence of renal insufficiency.^2^,^6^,^18^ However, there is bound to be some delay in diagnosis as they do not demonstrate the paraprotein in blood or urine which may shorten their survival.^6^ Hence a high index of suspicion should be kept in mind. FLC measurement seems to be a useful tool for monitoring and does not seem to be recommended as a screening method for nonsecretory multiple myeloma.^7^

In conclusion, absence of paraprotein in the blood does not exclude multiple myeloma. Though doing a bone marrow biopsy routinely in all cases with suspected multiple myeloma can put extra strain on the hematology department, we recommend consultation with hematologist when diagnosis of multiple myeloma is strongly suspected, in the absence of any abnormal protein in blood and urine, to avoid delay in diagnosis. We also suggest pooling of tissues of rare cancer in biobanks for future research. Molecular and genetic studies can be performed to help us understand their behavior.
